# DDB1 prepares brown adipocytes for cold-induced thermogenesis

**DOI:** 10.1093/lifemeta/loac003

**Published:** 2022-05-13

**Authors:** Xu Wang, Shen-Ying Liu, Guo-Sheng Hu, Hao-Yan Wang, Guo-Liang Zhang, Xiang Cen, Si-Ting Xiang, Wen Liu, Peng Li, Haobin Ye, Tong-Jin Zhao

**Affiliations:** State Key Laboratory of Genetic Engineering, Shanghai Key Laboratory of Metabolic Remodeling and Health, Institute of Metabolism and Integrative Biology, Zhongshan Hospital, Fudan University, Shanghai, China; Shanghai Qi Zhi Institute, Shanghai, China; State Key Laboratory of Genetic Engineering, Shanghai Key Laboratory of Metabolic Remodeling and Health, Institute of Metabolism and Integrative Biology, Zhongshan Hospital, Fudan University, Shanghai, China; School of Pharmaceutical Sciences, Fujian Provincial Key Laboratory of Innovative Drug Target Research, Xiamen University, Xiamen, Fujian, China; State Key Laboratory of Cellular Stress Biology, School of Life Sciences, Xiamen University, Xiamen, Fujian, China; State Key Laboratory of Cellular Stress Biology, School of Life Sciences, Xiamen University, Xiamen, Fujian, China; State Key Laboratory of Cellular Stress Biology, School of Life Sciences, Xiamen University, Xiamen, Fujian, China; State Key Laboratory of Genetic Engineering, Shanghai Key Laboratory of Metabolic Remodeling and Health, Institute of Metabolism and Integrative Biology, Zhongshan Hospital, Fudan University, Shanghai, China; School of Pharmaceutical Sciences, Fujian Provincial Key Laboratory of Innovative Drug Target Research, Xiamen University, Xiamen, Fujian, China; State Key Laboratory of Genetic Engineering, Shanghai Key Laboratory of Metabolic Remodeling and Health, Institute of Metabolism and Integrative Biology, Zhongshan Hospital, Fudan University, Shanghai, China; Shanghai Qi Zhi Institute, Shanghai, China; State Key Laboratory of Membrane Biology and Tsinghua-Peking Center for Life Sciences, School of Life Sciences, Tsinghua University, Beijing, China; State Key Laboratory of Genetic Engineering, Shanghai Key Laboratory of Metabolic Remodeling and Health, Institute of Metabolism and Integrative Biology, Zhongshan Hospital, Fudan University, Shanghai, China; State Key Laboratory of Genetic Engineering, Shanghai Key Laboratory of Metabolic Remodeling and Health, Institute of Metabolism and Integrative Biology, Zhongshan Hospital, Fudan University, Shanghai, China; Shanghai Qi Zhi Institute, Shanghai, China

**Keywords:** brown adipose tissue, thermogenesis, DDB1, RNA polymerase II, promoter-proximal pausing, UCP1, PGC1α

## Abstract

Brown adipose tissue (BAT) plays a key role in thermogenesis during acute cold exposure. However, it remains unclear how BAT is prepared to rapidly turn on thermogenic genes. Here, we show that damage-specific DNA binding protein 1 (DDB1) mediates the rapid transcription of thermogenic genes upon acute cold exposure. Adipose- or BAT-specific *Ddb1* knockout mice show severely whitened BAT and significantly decreased expression of thermogenic genes. These mice develop hypothermia when subjected to acute cold exposure at 4 °C and partial lipodystrophy on a high-fat diet due to deficiency in fatty acid oxidation. Mechanistically, DDB1 binds the promoters of *Ucp1* and *Ppargc1a* and recruits positive transcriptional elongation factor b (P-TEFb) to release promoter-proximally paused RNA polymerase II (Pol II), thereby enabling rapid and synchronized transcription of thermogenic genes upon acute cold exposure. Our findings have thus provided a regulatory mechanism of how BAT is prepared to respond to acute cold challenge.

## Introduction

The increasing prevalence of obesity and associated metabolic diseases worldwide has become a big challenge to public health, and new strategies are in urgent need to treat these diseases. Brown adipose tissue (BAT) is a major organ for non-shivering thermogenesis in mammals [1]. Upon cold exposure, BAT oxidizes metabolic fuels and generates heat via the activity of uncoupling protein 1 (UCP1) [2]. In rodent models, activation of BAT regulates glucose homeostasis and insulin sensitivity by increasing glucose and lipid clearance [3–5]. In many mouse models, enhanced BAT activity leads to resistance to weight gain [6]. Adult humans have metabolic active BAT [7–11]. Activated BAT is associated with accelerated lipid metabolism and improved insulin sensitivity [12–14]. Therefore, targeting BAT represents a promising strategy to treat metabolic diseases [3].

The thermogenesis of BAT is controlled at multiple levels [6]. Upon cold exposure, sympathetic nerves release norepinephrine to activate PKA in brown adipocytes, which phosphorylates CREB and ATF2 to activate the transcription of *Ucp1* and *Ppargc1a* (encoding PGC1α) [15]. PGC1α then cooperates with other transcriptional factors to induce the transcription of thermogenic genes including *Ucp1* [6]. Other epigenetic and transcriptional factors also play essential roles in the transcription of thermogenic genes [16, 17]. However, it remains unclear how the thermogenic genes are rapidly transcribed upon cold challenge.

Promoter-proximal pausing of RNA polymerase II (Pol II) is a regulatory mechanism for transcription of immediate early response genes involved in stimulus-responsive pathways [18, 19]. In Pol II pausing, the pre-initiation complex is formed, but Pol II pauses after a synthesis of 20–60 nt of mRNA [20]. In response to various stimuli or developmental cues, the positive transcription elongation factor b (P-TEFb) is released from its inhibitory complex, phosphorylates Pol II at Ser2 of its C-terminal repeat, and activates Pol II, thereby enabling rapid and synchronized expression of the downstream genes [21, 22]. Emerging roles of Pol II pausing have been demonstrated in mammalian embryonic stem cells [23–25], but not much is known in other cell lineages.

Damage-specific DNA binding protein 1 (DDB1) is well recognized as a component of the Cullin4 (CUL4)-RING E3 ubiquitin ligase complex that regulates a variety of physiological events using a subset of WD40 proteins as adaptors [26–28]. Recently, we showed that DDB1 works both in CUL4-dependent and CUL4-independent manner to regulate adipogenesis [29, 30]. In the very early stage of adipogenesis, DDB1 works independently of CUL4 by recruiting P-TEFb to the immediate early response genes to initiate the transcriptional cascade [29]. In the late stage, DDB1 complexes with CUL4 and WDTC1 to ubiquitinate and degrade MED20, a subunit of the Mediator complex, to inhibit adipogenesis [30]. Here, to investigate the role of DDB1 in mature adipocytes, we crossed *Ddb1*^*f/f*^ mice with *AdipoQ-Cre* or *Ucp1-Cre* mice and generated adipose- or BAT-specific knockout mice of *Ddb1*. We characterized the mice and found that depletion of DDB1 in BAT greatly disrupted the thermogenic function. We showed that the thermogenic genes were subjected to regulation by promoter-proximal pausing of Pol II and that DDB1 was required for the release of paused Pol II. Our studies provide a mechanism for how thermogenic genes are rapidly turned on upon acute cold exposure.

## Results

### DDB1 is required for the maintenance of the brown phenotype of BAT

**Figure 1:**
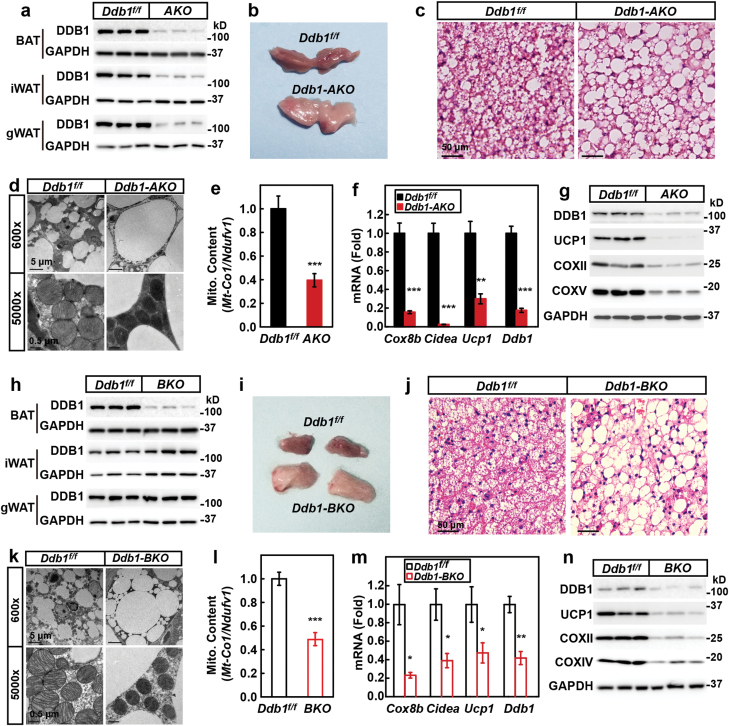
DDB1 is required for the maintenance of the brown phenotype of BAT. Male (12 weeks old) *Ddb1*^*f/f*^ (a–n), *AdipoQ-Cre*–*Ddb1*^*f/f*^ (*Ddb1-AKO*) (a–g), and *Ucp1-Cre*–*Ddb1*^*f/f*^ (*Ddb1-BKO*) (h–n) mice were used in the experiments. (a, h) Analysis of DDB1 protein levels in BAT, iWAT, and gWAT of *Ddb1-AKO* (a) and *Ddb1-BKO* (h) mice. (b, c, i, j) Images and H&E staining of the BAT from control and *Ddb1-AKO* (b, c), or *Ddb1-BKO* (i, j) mice. Scale bar, 50 μm. (d, k) Electron microscopy analysis of the BAT from control and *Ddb1-AKO* (d), or *Ddb1-BKO* (k) mice. Scale bars are as indicated. (e, l) Analysis of the mitochondrial DNA content by qRT-PCR in control and *Ddb1-AKO* (e), or *Ddb1-BKO* (l) mice. The level of mtDNA in control mice was normalized to 1.0. (f, m) qRT-PCR analysis of the mRNA levels of indicated genes; *36B4* was used as invariant control. The expression level of each gene in control mice was normalized to 1.0. Each value represents the mean ± SEM of four mice. Asterisks (*) denote the level of statistical significance (Student’s *t*-test) between control and *Ddb1-AKO* or *Ddb1-BKO* mice. *P < .05; **P < .01; ***P < .001. (g, n) Western blot analysis of indicated proteins in BAT of control and *Ddb1-AKO* (g), or *Ddb1-BKO* (n) mice.

To explore whether and how DDB1 regulates the physiological function of mature adipocytes, we crossed *Ddb1*^*f/f*^ mice with *AdipoQ-Cre* mice and generated adipocyte-specific *Ddb1* knockout mice, designated as *Ddb1-AKO*. DDB1 was largely depleted in BAT, inguinal WAT (iWAT), and gonadal WAT (gWAT) of *Ddb1-AKO* mice (Fig. 1a). As shown in Fig. 1b, the BAT of *Ddb1-AKO* mice appeared severely whitened compared with that in *Ddb1*^*f/f*^ mice. Hematoxylin and eosin (H&E) analysis revealed that the lipid droplets were dramatically enlarged in the BAT of *Ddb1-AKO* mice (Fig. 1c). A close examination by electron microscopy revealed that DDB1-deficient brown adipocytes exhibited not only enlarged lipid droplets but also smaller mitochondria that appeared to be darker and contained less cristae (Fig. 1d). Loss of DDB1 also significantly decreased the mitochondrial DNA content in BAT (Fig. 1e). Furthermore, both mRNA and protein levels of BAT marker genes were significantly decreased in the BAT of *Ddb1-AKO* mice (Fig. 1f and g).

We have also examined the adipogenesis markers in the BAT of *Ddb1*^*f/f*^ and *Ddb1-AKO* mice. As shown in Supplementary Fig. S1a, there was not much difference in the protein levels of PERILIPIN, PPARγ, C/EBPα, and CD36. The DNA content of BAT was also not different between the two strains (Supplementary Fig. S1b). These data indicate that the phenotype of BAT in *Ddb1-AKO* mice is not due to defects in adipogenesis.

To ensure that the whitening of BAT in the *Ddb1-AKO* was not secondary to changes in WAT, we crossed *Ddb1*^*f/f*^ mice with *Ucp1-Cre* mice and generated BAT-specific *Ddb1* knockout mice (*Ddb1-BKO*) (Fig. 1h). Very similar to *Ddb1-AKO* mice, *Ddb1-BKO* mice showed whitened BAT with enlarged lipid droplets and less mitochondrial content (Fig. 1i–l). The expression levels of BAT-specific genes were largely reduced in the BAT of *Ddb1-BKO* mice (Fig. 1m and n). These results indicate that DDB1 maintains the brown phenotype of BAT in a cell-autonomous manner.

**Figure 2:**
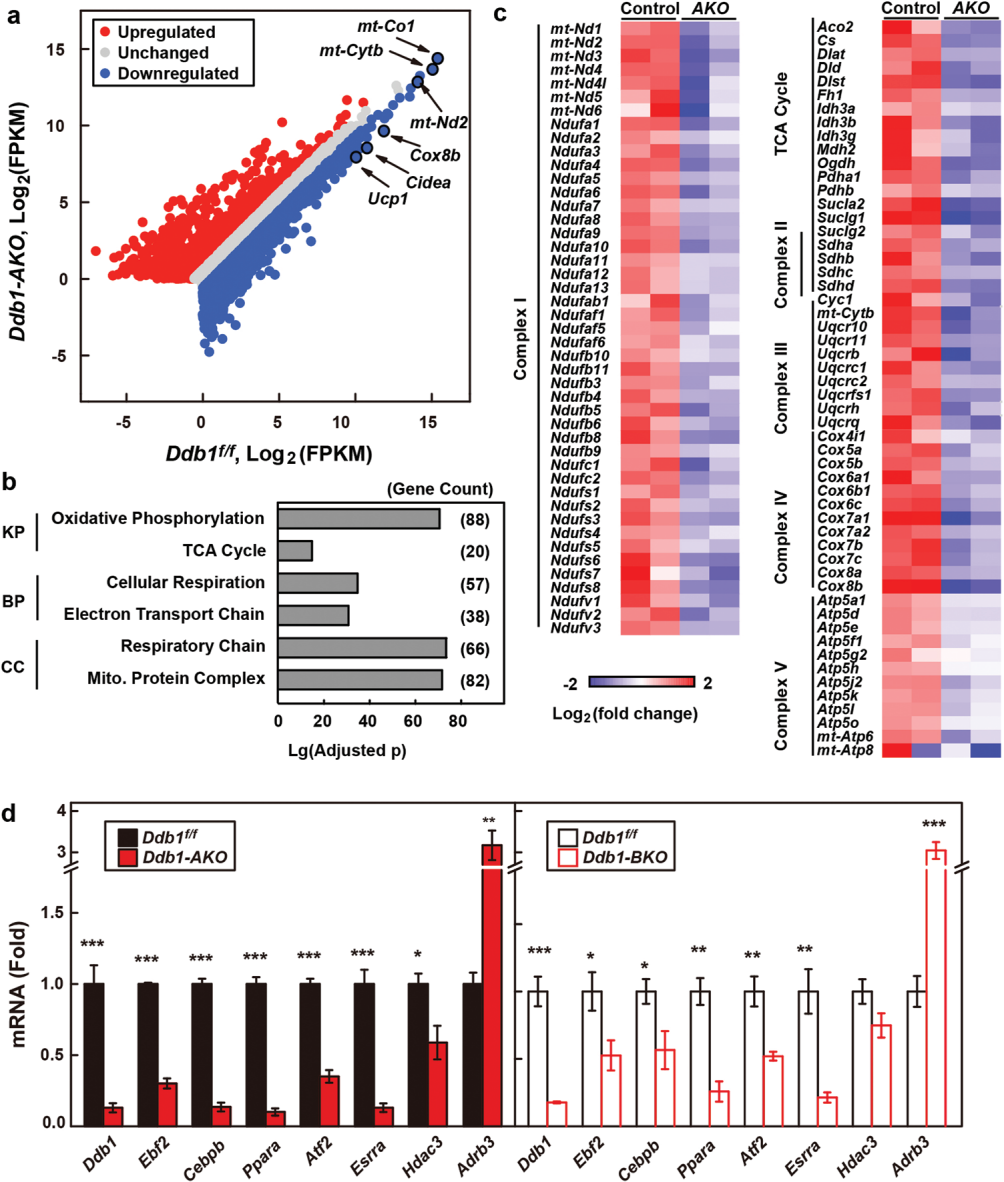
DDB1 is required for the expression of thermogenic genes in BAT. (a–c) RNA-seq analysis of the BAT of *Ddb1-AKO* and control littermates (12-week-old male). Each sample contained RNAs pooled from three mice of each genotype. (a) The DDB1-regulated genes are shown in a scatter plot with a fold change greater than 1.5. FPKM, fragments per kilobase per million. Genes with an FPKM value greater than 1.0 were included in the analysis. (b) Gene ontology and pathway analysis of the downregulated genes in the BAT of *Ddb1-AKO* mice. KP, KEGG pathway; BP, biological process; CC, cellular component. (c) Heat map analysis of the genes of mitochondrial complexes I–V and TCA cycles. (d) qRT-PCR analysis of the representative thermogenic genes in control and *Ddb1-AKO* (left), or *Ddb1-BKO* (right) mice. Each value represents the mean ± SEM of four mice. Asterisks (*) denote the level of statistical significance (Student’s *t-*test) between control and *Ddb1-AKO* or *Ddb1-BKO* mice. *P < .05; **P < .01; ***P < .001.

To further evaluate the effect of DDB1 on BAT, we extracted mRNA from BAT of *Ddb1-AKO* and their littermate controls and performed RNA sequencing (RNA-seq). Among the 12,290 genes analyzed, 1827 genes were downregulated in the *Ddb1-AKO* mice by more than 1.5-fold, including the BAT-specific genes, such as *Cox8b*, *Cidea*, and *Ucp1*, and the mitochondrial genes, such as *mt-Co1* and *mt-Cytb* (Fig. 2a). Gene ontology analysis of the suppressed genes in the *Ddb1-AKO mice* revealed a strong enrichment of genes involved in mitochondrial oxidative phosphorylation, tricarboxylic acid (TCA) cycle, cellular respiration, electron transport chain, respiratory chain, and mitochondrial protein complex (Fig. 2b). A close examination revealed that almost all the genes of mitochondrial complex I–V were downregulated in the BAT of *Ddb1-AKO* mice (Fig. 2c), which was further confirmed by quantitative real-time polymerase chain reaction (qRT-PCR) analysis (Supplementary Fig. S1d). In contrast, glycolysis genes did not show much difference between *Ddb1*^*f/f*^ and *Ddb1-AKO* mice (Supplementary Fig. S1c). Furthermore, some of the key transcriptional regulators of BAT, including *Ebf2*, *Cebpb*, *Ppara*, *Atf2*, *Esrra*, and *Hdac3* [6, 31, 32], were significantly decreased in both *-Ddb1-AKO* and *Ddb1-BKO* mice (Fig. 2d). The mRNA level of *Adrb3* was significantly increased in DDB1-deficient BAT (Fig. 2d), implying a compensatory effect. These data suggest that DDB1 might be a master transcriptional regulator of thermogenic genes.

### DDB1 is required for cold-induced thermogenesis

**Figure 3:**
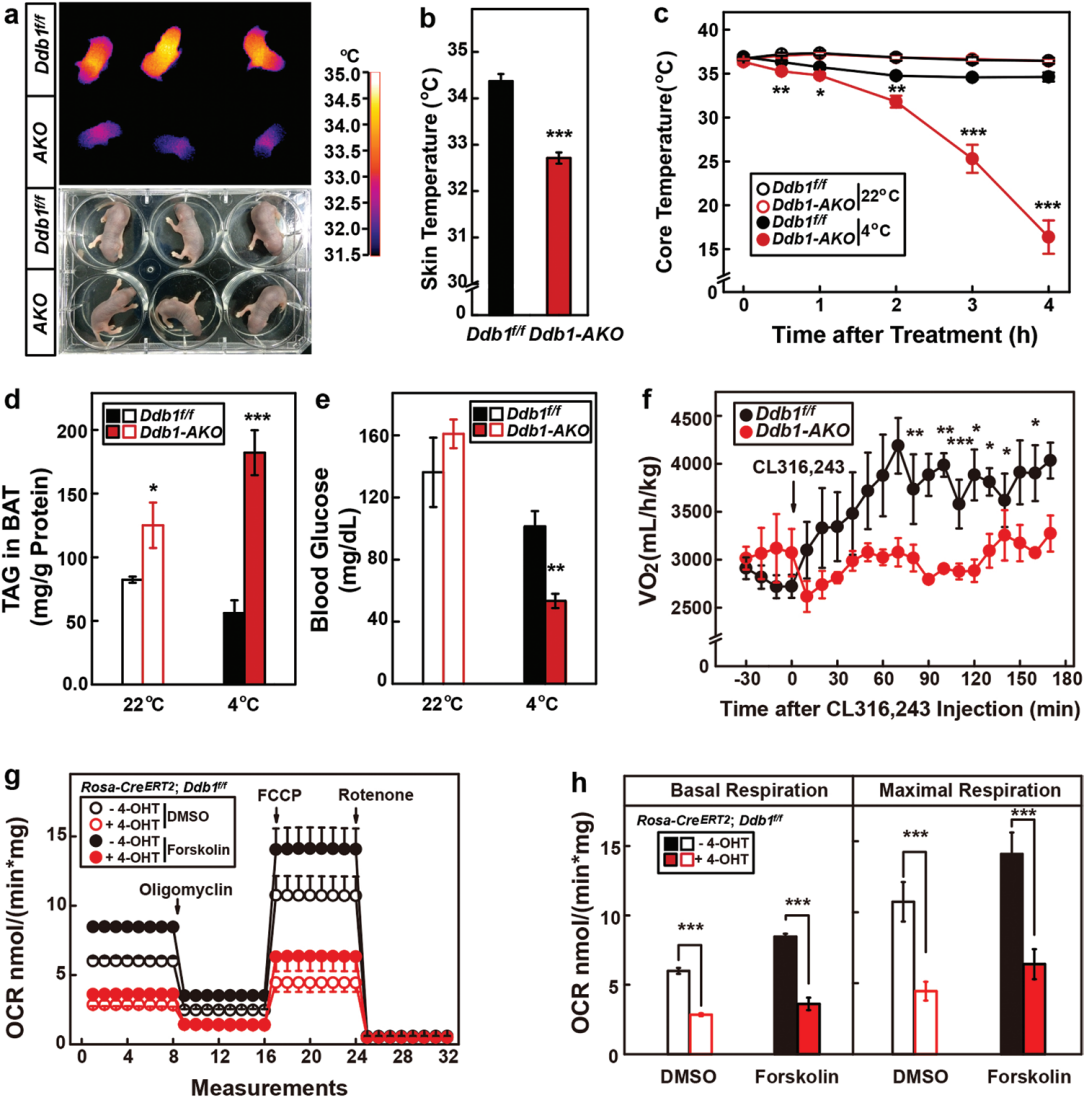
DDB1 is required for cold-induced thermogenesis. (a, b) Infrared imaging of pups (postnatal day 3) of *Ddb1-AKO* and their control littermates. Photographic images of the pups are shown in the lower panels in (a). The skin temperatures from the infrared images were quantified and shown in (b). Each value represents the mean ± SEM of three mice. Asterisks (*) denote the level of statistical significance (Student’s *t*-test). ***P < .001. (c–e) Single-housed *Ddb1-AKO* and control littermates (12-week-old male) were either maintained at 22 °C or subjected to cold exposure at 4 °C for 4 h. Their core temperatures were measured at the indicated times (c). At the end of the experiment, mice were euthanized, and the triglyceride content in BAT (d) and blood glucose (e) was measured. Each value represents the mean ± SEM of six mice. Asterisks (*) denote the level of statistical significance (Student’s *t*-test). *P < .05; **P < .01; ***P < .001. (f) *Ddb1-AKO* and control littermates (8-week-old male) received an intraperitoneal injection of CL316,243 (10 mg/kg), and the oxygen consumption of each mouse was monitored by metabolic cage for 3 h. Each value represents the mean ± SEM of four mice. Asterisks (*) denote the level of statistical significance (Student’s *t*-test). *P < .05; **P < .01; ***P < .001. (g, h) Primary SVFs were isolated from BAT of *Rosa-Cre*^*ERT2*^–*Ddb1*^*f/f*^ mice and subject to differentiation. Cells were treated with 8 μM 4-OHT from day 4 to 8 to induce the deletion of *Ddb1*. On day 8 of differentiation, cells were subjected to O_2_K analysis in the presence or absence of forskolin (10 μM). OCR was recorded after indicated treatments (g) and quantified in (h). Each value represents the mean ± SEM of three samples. Asterisks (*) denote the level of statistical significance (Student’s *t*-test). ***P < .001.

We then directly tested the role of DDB1 in the thermogenic functions of BAT. First, we examined the body temperature of pups of *Ddb1-AKO* and control littermates on postnatal day 3. As shown in Fig. 3a and b, the skin temperature of the *Ddb1-AKO* mice (32.7 ± 0.1 °C) was significantly lower than that (34.3 ± 0.2 °C) of the control littermates.

We then studied the role of DDB1 in adult mice. When *Ddb1*^*f/f*^ and *Ddb1-AKO* mice were housed at 22 °C, both strains could maintain their core temperatures around 37 °C (Fig. 3c). When they were switched to 4 °C, the *Ddb1*^*f/f*^ mice could maintain their core temperatures around 35 °C; however, the *Ddb1-AKO* mice failed to do so, and their core temperatures decreased to 16.4 ± 1.9 °C after 4 h of cold exposure (Fig. 3c). At this point, the *Ddb1-AKO* mice became moribund and we had to stop the experiment. After the 4-h cold exposure, the triglyceride content in BAT was higher (Fig. 3d), but the blood glucose level was significantly lower (Fig. 3e) in the *Ddb1-AKO* mice, indicating an impaired utilization of fatty acids and increased utilization of glucose.

To further test the function of BAT in response to cold challenge, we treated mice with CL316,243, an agonist of the β3-adrenergic receptor, and monitored oxygen consumption in a metabolic cage. While *Ddb1*^*f/f*^ mice exhibited increased oxygen consumption after CL316,243 injection, *Ddb1-AKO* mice barely showed any response (Fig. 3f), which further confirms that lack of DDB1 results in dysfunctional BAT.

We then isolated primary stromal vascular fractions (SVFs) from BAT of *Rosa-Cre*^*ERT2*^–*Ddb1*^*f/f*^ mice, and induced differentiation into brown adipocytes, followed by treatment with 4-hydroxytamoxifen (4-OHT) to induce deletion of *Ddb1* and analysis of oxygen consumption rate (OCR). As shown in Fig. 3g and h, the OCR in DDB1-deficient cells was significantly lower than that in control cells under both basal and forskolin-stimulated conditions.

We next performed the same experiments in *Ddb1-BKO* mice. Consistent with the results obtained from *Ddb1-AKO* mice, both pups and adults of *Ddb1-BKO* mice had defects in thermogenesis in response to cold challenge (Supplementary Fig. S2a–f), which further confirms the critical role of DDB1 in cold-induced thermogenesis.

We have also examined the role of DDB1 in the browning of iWAT. As shown in Supplementary Fig. S3a and b, after 10 consecutive days of administration of CL316,243, both mRNA and protein levels of *Ucp1*, *Ppargc1a*, and the mitochondrial genes were dramatically upregulated in the iWAT of *Ddb1*^*f/f*^ mice; however, such effect was largely blocked in *Ddb1-AKO* mice. H&E staining and immunostaining using anti-UCP1 antibody revealed that mice lacking DDB1 showed less extent of browning with dramatically reduced the expression of UCP1 (Supplementary Fig. S3c).

### Loss of DDB1 in BAT disrupts whole-body lipid metabolism

**Figure 4:**
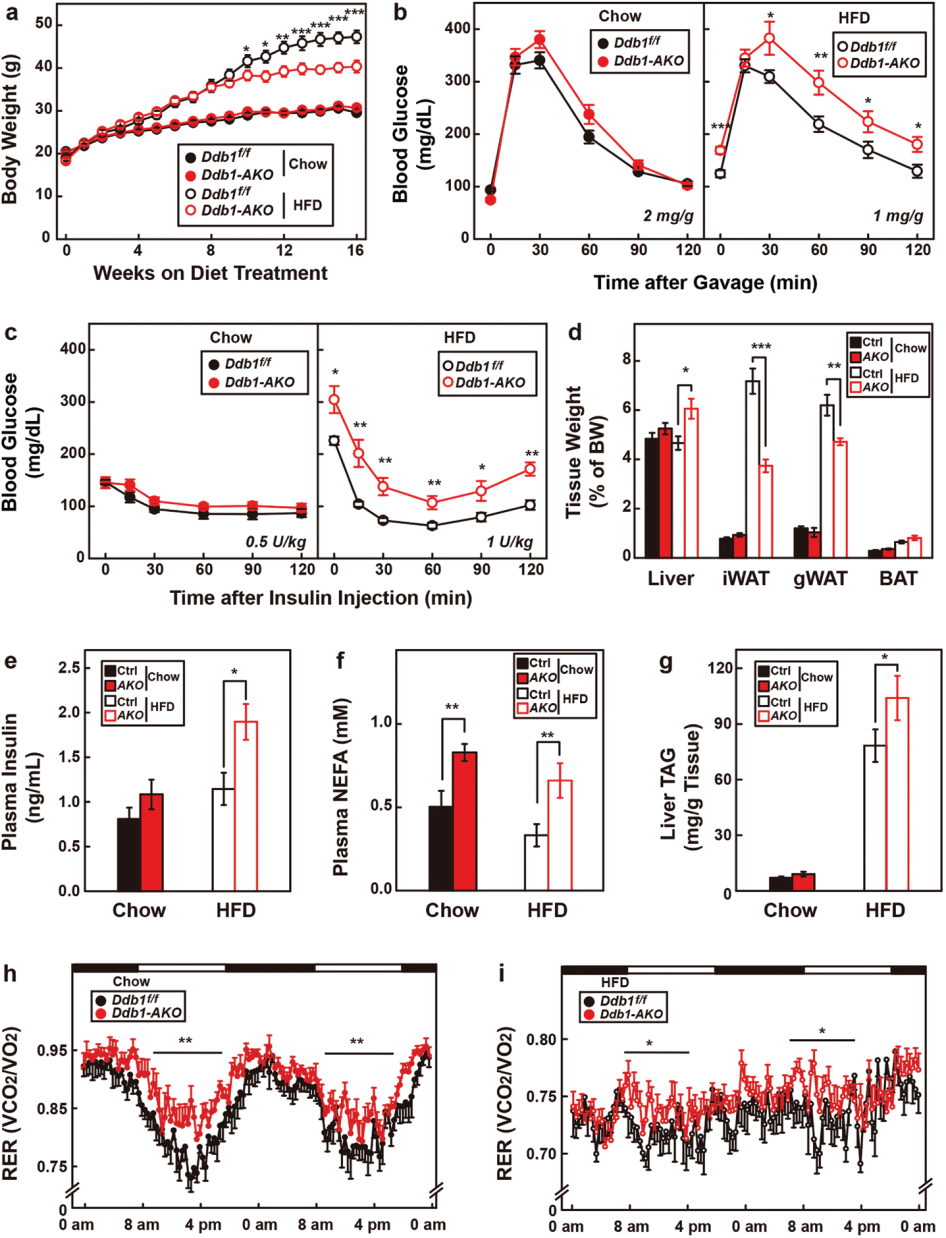
*Ddb1-AKO* mice develop partial lipodystrophy on HFD feeding. (a–i) *Ddb1-AKO* and control littermates (4-week-old male, *n* = 8 per group) were subjected to chow or HFD feeding for 16 weeks. (a) Body weight was monitored every week throughout the whole period. (b) On week 10, after a 16-h fast, each mouse received an oral gavage of glucose at a dose of 2 and 1 mg/g body weight for mice on chow and HFD, respectively. Blood glucose was measured from the tail vein at the indicated time. (c) On week 12, after a 4-h fast, each mouse received an intraperitoneal injection of insulin at a dose of 0.5 and 1 U/kg for mice on chow and HFD, respectively. Blood glucose was measured from the tail vein at indicated times. (d–g) On week 16, mice were sacrificed and indicated tissues were collected and the ratios of tissue weight/body weight were plotted in (d). Plasma insulin (e), plasma-free fatty acids (f), and liver triglyceride (g) were measured. Each value represents the mean ± SEM of eight mice. (h, i) Chow (h) and HFD-fed (i) mice were subjected to metabolic cage analysis. Respiratory exchange ratios for two consecutive days were plotted. Each value represents the mean ± SEM of six mice. Each asterisk (*) denotes the level of statistical significance (Student’s *t*-test) between *Ddb1-AKO* and *Ddb1*^*f/f*^ mice. *P < .05; **P < .01; ***P < .001.

We then sought to examine the effect of whitened BAT on whole-body metabolic homeostasis. We first subjected *Ddb1-AKO* mice and their control littermates to chow and high-fat diet (HFD) for 16 weeks. While *Ddb1*^*f/f*^ and *Ddb1-AKO* mice showed no difference in their body weight on chow diet, the body weight of HFD-fed *Ddb1-AKO* mice was significantly lower than *Ddb1*^*f/f*^ mice starting from week 10 (Fig. 4a). However, the HFD-fed *Ddb1-AKO* mice showed decreased capability to clear glucose (Fig. 4b), and they were insulin resistant (Fig. 4c). On week 16 of HFD feeding, *Ddb1-AKO* mice showed higher liver weight and decreased weights of iWAT and gWAT (Fig. 4d). The *Ddb1-AKO* mice showed higher plasma levels of insulin (Fig. 4e) and free fatty acids (Fig. 4f) and significantly elevated liver triglyceride content (Fig. 4g), resembling the phenotype of partial lipodystrophy.

To further characterize these mice, we subjected them to metabolic cage analysis. As shown in Fig. 4h and i, the respiratory exchange ratio of *Ddb1-AKO* mice during daytime was significantly higher than that in *Ddb1*^*f/f*^ mice under both chow- and HFD-fed conditions, indicating that *Ddb1-AKO* mice have a defect in utilizing fatty acids to maintain body temperature. These results were supported by higher levels of plasma-free fatty acids in *Ddb1-AKO* mice (Fig. 4f) and further explained the ectopic lipid storage in the liver (Fig. 4g).

We have also performed the same experiments in *Ddb1-BKO* mice and their control littermates. Similarly, *Ddb1-BKO* mice also gained less weight on HFD but developed insulin resistance and partial lipodystrophy (Supplementary Fig. S4a–g), confirming that loss of DDB1 in BAT disrupts whole-body lipid metabolism.

### DDB1 binds promoters of early response thermogenic genes to control their transcription

We then went on to interrogate the underlying mechanism of how DDB1 controls thermogenesis in BAT. DDB1 typically functions as a component of the CUL4 E3 ligase complex [27], but we have previously shown that DDB1 can also act in a CUL4-independent manner [29]. To examine whether the function of DDB1 in regulating thermogenesis was dependent on the CUL4 E3 ligase complex, we crossed *Cul4a*^*f/f*^ and *Cul4b*^*f/y*^ mice with *AdipoQ-Cre* mice to generate adipose tissue-specific knockout of *Cul4a* (*Cul4a-AKO*) and *Cul4b* (*Cul4b-AKO*) mice, respectively. Supplementary Fig. S5a–c and e–g shows that neither *Cul4a-AKO* nor *Cul4b-AKO* mice showed any defect in BAT morphology or expression of BAT-specific genes. And these mice showed no difference from littermate controls in response to acute cold exposure (Supplementary Fig. S5d and h). Therefore, DDB1 might function in a CUL4-independent manner to regulate the thermogenesis of BAT.

**Figure 5:**
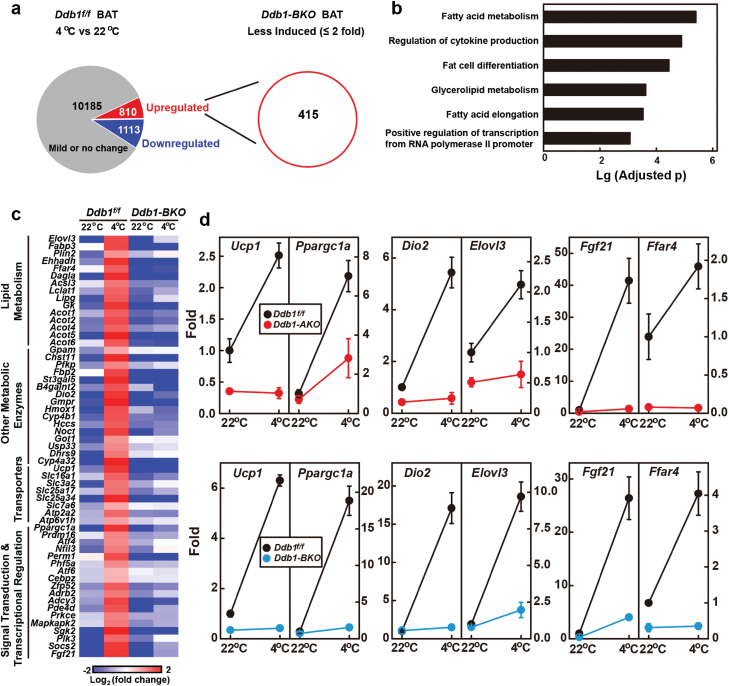
DDB1 is required for the expression of cold-induced thermogenic genes. (a, b) Single-housed *Ddb1-BKO* and control littermates (12-week-old male) were kept at 22 °C or subjected to cold exposure at 4 °C for 4 h. BAT was collected from each mouse, and the total RNA was extracted. For each treatment, RNA from three mice was pooled and subjected to RNA-Seq analysis. (b) DDB1 downstream genes were subjected to gene ontology analysis. (c) Heat map analysis of the representative genes. (d) qRT-PCR analysis of the mRNA levels of *Ucp1*, *Ppargc1a*, *Dio2*, *Elvol3*, *Fgf21*, and *Ffar4* in BAT of *Ddb1*^*f/f*^, *Ddb1-AKO*, and *Ddb1-BKO* mice in response to acute cold exposure. Each value represents the mean ± SEM of three mice.

We then explored whether DDB1 would directly regulate the transcription of thermogenic genes. We exposed *Ddb1*^*f/f*^ and *Ddb1-BKO* mice to 4 °C for 4 h, extracted mRNAs from BAT, and subjected them to RNA-Seq analysis. In *Ddb1*^*f/f*^ mice, 810 genes were early response genes that were upregulated more than 2-fold by cold exposure (Fig. 5a). Among them, the fold induction of 415 genes in *Ddb1-BKO* mice was less than 50% of that in *Ddb1*^*f/f*^ mice, and they were designated as DDB1 downstream genes (Fig. 5a). Gene ontology analysis revealed that these genes were involved in fatty acid metabolism, regulation of cytokine production, fat cell differentiation, glycerolipid metabolism, fatty acid elongation, and positive regulation of transcription from Pol II promoter (Fig. 5b). Fig. 5c shows the heat map of some of the representative genes. To further confirm the results, we performed a qRT-PCR analysis and found that cold induction of *Ucp1*, *Ppargc1a*, *Dio2*, *Elovl3*, *Fgf21*, and *Ffar4* was largely blunted in the BAT of both *Ddb1-AKO* and *Ddb1-BKO* mice (Fig. 5d).

**Figure 6:**
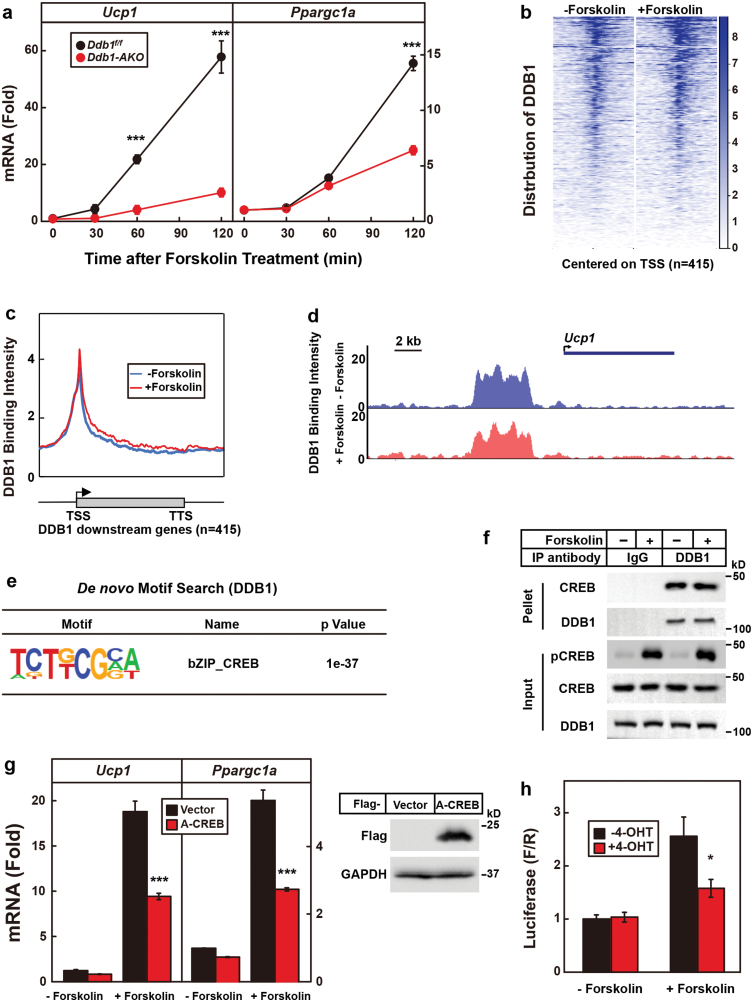
DDB1 binds to the promoters of the thermogenic genes. (a) Primary SVFs were isolated from BAT of *Ddb1*^*f/f*^ and *Ddb1-AKO* mice and differentiated into mature brown adipocytes. On day 8 of differentiation, cells were treated with forskolin (10 μM) for indicated time before harvest for qRT-PCR analysis of *Ucp1* and *Ppargc1a*. *Cyclophilin* was used as an invariant control. Each value represents the mean ± SEM of three samples. Asterisks (*) denote the level of statistical significance (Student’s *t*-test). ***P < .001. (b–e) ChIP-Seq was performed in SVF-derived brown adipocytes (*Ddb1*^*f/f*^) in the presence or absence of forskolin (10 μM) for 1 h. (b) Heat map analysis of the distribution of DDB1 on its downstream genes. (c) Global analysis of the binding intensity of DDB1 on its downstream genes. (d) Binding of DDB1 on the promoter of *Ucp1*. (e) *De novo* motif search of DDB1-binding sites. (f) SVF-derived brown adipocytes were lysed and subjected to immunoprecipitation using IgG or anti-DDB1 antibody. Input and pellet fractions were analyzed by Western blot using indicated antibodies. (g) SVF-derived brown adipocytes were infected with retrovirus expressing a dominant-negative form of CREB (A-CREB). Cells were then treated with or without forskolin (10 μM) for 2 h and harvested for qRT-PCR analysis of *Ucp1* and *Ppargc1a*. Cyclophilin was used as an invariant control. Each value represents the mean ± SEM of three samples. Asterisks (*) denote the level of statistical significance (Student’s *t*-test). ***P < .001. A small portion of the cells were subjected to Western blot to indicate the expression of A-CREB. (h) A CRE-reporter plasmid (750 ng) and a *Renilla* luciferase plasmid (3 ng) were transfected into control and DDB1-deficient (4-OHT) brown adipocytes as described in the Methods section. Cells were treated with or without forskolin (10 μM) for 8 h before harvest for dual luciferase reporter assay. Each value represents the mean ± SEM of three samples. Asterisks (*) denote the level of statistical significance (Student’s *t*-test). *P < .05.

To directly test the effect of DDB1 on the transcription of its downstream genes, we isolated primary SVFs from BAT of *Ddb1*^*f/f*^ and *Ddb1-AKO* mice, induced differentiation into mature adipocytes, and treated with forskolin. As shown in Fig. 6a, *Ucp1* and *Ppargc1a* were dramatically induced by forskolin in *Ddb1*^*f/f*^ cells, but such induction was significantly reduced in DDB1-deficient cells.

We then performed ChIP-Seq to visualize the binding of DDB1 on its downstream genes. Indeed, DDB1 bound the promoters of its downstream genes, and its binding intensity was not changed by forskolin treatment (Fig. 6b and c). Fig. 6d shows that DDB1 directly bound the promoter of *Ucp1*. *De novo* motif search revealed that DDB1-binding sites overlapped with that of bZIP_CREB family of transcription factors (Fig. 6e), which has been shown to play a critical role in thermogenesis [6, 33]. Immunoprecipitation assay revealed that endogenous DDB1 interacted with CREB in the presence or absence of forskolin (Fig. 6f).

Furthermore, when A-CREB, a dominant-negative form of CREB, was overexpressed in brown adipocytes, it indeed significantly decreased forskolin-induced expression of *Ucp1* and *Ppargc1a* (Fig. 6g). We then performed dual luciferase reporter assay and found that depletion of DDB1 significantly decreased the CRE-reporter activity in response to forskolin (Fig. 6h), indicating that DDB1 directly regulates the transcriptional activity of CREB.

### DDB1 facilitates the release of paused Pol II on the thermogenic genes

**Figure 7:**
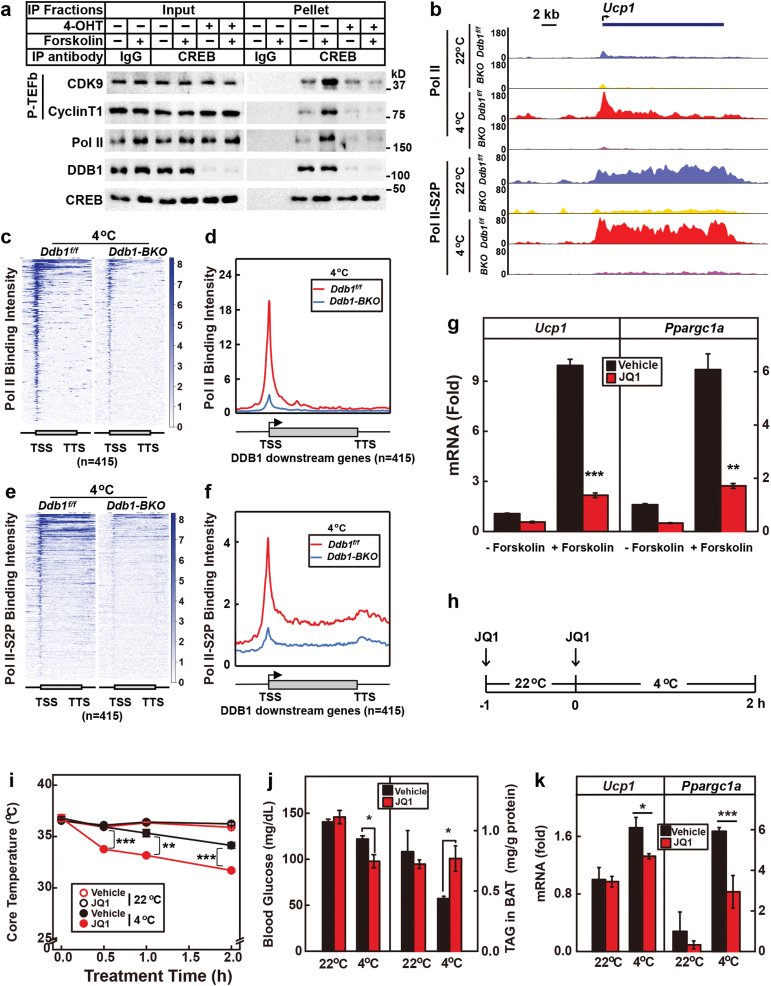
DDB1 promotes the release of paused Pol II on its downstream genes. (a) Control and DDB1-deficient (4-OHT) brown adipocytes were subjected to immunoprecipitation using IgG or anti-CREB antibody. Input and pellet fractions were subjected to Western blot using indicated antibodies. (b–f) *Ddb1*^*f/f*^ and *Ddb1-BKO* mice were maintained at 22 °C or subjected to acute cold exposure at 4 °C for 4 h as in Fig. 2c. BAT was collected, homogenized, fixed, and subjected to ChIP-Seq analysis using anti-Pol II and anti-Pol II-S2P antibodies as described in the Methods section. (b) Binding profiles of Pol II and Pol II-S2P on *Ucp1* gene. (c–f) Heat map and binding intensity analysis of Pol II (c, d) and Pol II-S2P (e, f) on the 415 downstream genes of DDB1 in the BAT of *Ddb1*^*f/f*^ and *Ddb1-BKO* mice at 4 °C. (g) SVF-derived brown adipocytes (*Ddb1*^*f/f*^) were pretreated with vehicle or JQ1 (0.5 μM) and then treated with or without forskolin (10 μM) for 2 h before harvest for qRT-PCR analysis of *Ucp1* and *Ppargc1a*. Cyclophilin was used as an invariant control. Each value represents the mean ± SEM of three samples. Asterisks (*) denote the level of statistical significance (Student’s *t*-test). **P < .01; ***P < .001. (h–k) Single-housed WT C57BL6 mice (male, *n* = 3) were injected with vehicle or JQ1 (50 mg/kg) −1 and 0 h before cold exposure. Mice were then maintained at 22 °C or subjected to a 4 °C cold challenge for 2 h. (i) Core temperatures were measured at the indicated time. (j) Blood glucose and BAT content of triglyceride were measured at the end of the experiment. (k) mRNA was extracted from BAT for qRT-PCR analysis of *Ucp1* and *Ppargc1a*. Cyclophilin was used as an invariant control. Each value represents the mean ± SEM of three mice. Asterisks (*) denote the level of statistical significance (Student’s *t*-test). *P < .05; **P < .01; ***P < .001.

We next sought to know how DDB1 controls the transcription of CREB downstream thermogenic genes. As we have previously shown that DDB1 recruits P-TEFb to turn on the immediate early response genes in adipogenesis [29], we examined whether DDB1 would function in a similar way to control the transcription of the early response thermogenic genes. We first performed immunoprecipitation using the anti-CREB antibody in control and DDB1-deficient brown adipocytes. Fig. 7a shows that immunoprecipitation of CREB pulled down Pol II and the two subunits of P-TEFb, CDK9 and Cyclin T1, and treatment with forskolin enhanced their interaction. However, when DDB1 was depleted from these cells, the interaction between CREB and Pol II or P-TEFb was largely abolished (Fig. 7a), indicating a critical role of DDB1 in recruiting P-TEFb to CREB downstream genes.

To directly test whether the early response thermogenic genes were regulated by Pol II pausing and to study the role of DDB1 in the process, we performed ChIP-Seq analysis of Pol II and Pol II-S2P in BAT of *Ddb1*^*f/f*^ and *Ddb1-BKO* mice at 22 °C or 4 °C. Pol II-S2P is the transcriptionally active form of Pol II that is phosphorylated by P-TEFb at Ser2 in its C-terminal repeats [34]. At 22 °C, Pol II bound the proximal promoter of *Ucp1* in the BAT of both *Ddb1*^*f/f*^ and *Ddb1-BKO* mice but showed a higher binding intensity on the gene body in *Ddb1*^*f/f*^ mice (Fig. 7b). Consistently, the binding of Pol II-S2P on the gene body of *Ucp1* is much higher in *Ddb1*^*f/f*^ mice (Fig. 7b). When the mice were subjected to acute cold exposure, the binding of both Pol II and Pol II-S2P dramatically increased in *Ddb1*^*f/f*^ mice but not in *Ddb1-BKO* mice (Fig. 7b). Similar observations were made on the binding of Pol II and Pol II-S2P on *Ppargc1a* (Supplementary Fig. S6a). We have also performed a global analysis of Pol II and Pol II-S2P binding on the 415 DDB1 downstream genes and found that DDB1 was indeed required for the binding of Pol II and Pol II-S2P on the proximal promoters of the early response thermogenic genes (Fig. 7c–f; Supplementary Fig. S6b–e). These results indicate that the early response thermogenic genes are subject to regulation by Pol II pausing and that DDB1 plays an essential role in the release of paused Pol II.

To further confirm that the early response thermogenic genes are regulated by promoter-proximal pausing of Pol II, we treated SVF-derived brown adipocytes with JQ1, an inhibitor of BRD4, to block the recruitment of P-TEFb and release of paused Pol II [35, 36]. Fig. 7g shows that treatment with JQ1 significantly decreased forskolin-induced transcription of *Ucp1* and *Ppargc1a*. We then pretreated wild type (WT) mice with JQ1 followed by acute cold exposure at 4 °C and found that JQ1-treated mice showed significantly lower core temperatures (Fig. 7h and i). These mice also showed decreased blood glucose levels and increased BAT triglyceride content after cold exposure (Fig. 7j). Cold-induced transcription of *Ucp1* and *Ppargc1a* was significantly reduced by JQ1 treatment (Fig. 7k). These data provided further evidence that cold-induced thermogenesis is regulated by Pol II pausing.

**Figure 8:**
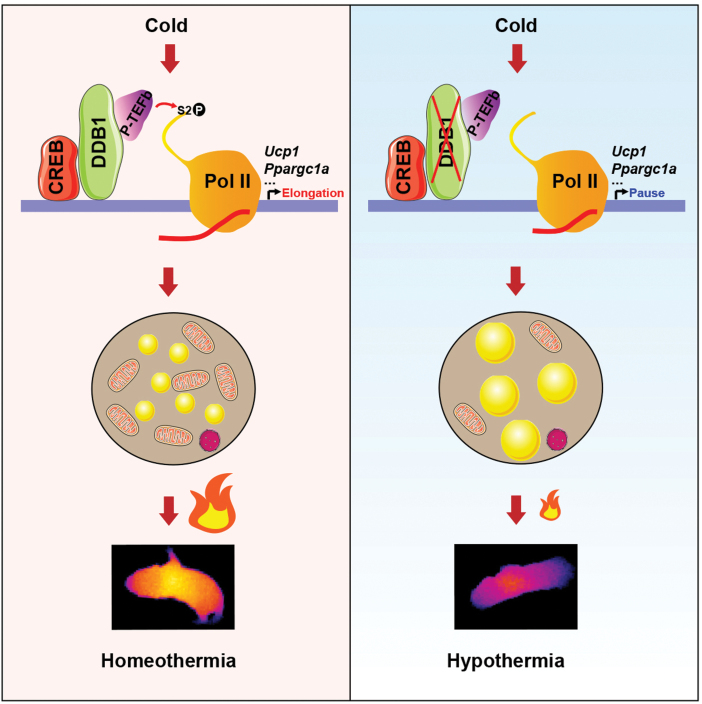
A working model for how DDB1 prepares BAT for acute cold challenge. When exposed to acute cold challenge, DDB1 recruits P-TEFb to the proximal promoters of the thermogenic gens including *Ucp1* and *Ppargc1a* in brown adipocytes. P-TEFb then phosphorylates paused Pol II at Ser2 and activates Pol II. The release of paused Pol II re-initiates transcriptional elongation of the thermogenic genes, which prepares the mice for acute cold challenge. In DDB1-deficient cells, P-TEFb is not efficiently recruited to the thermogenic genes upon cold challenge, resulting in decreased expression of these genes. DDB1-deficient mice thus develop hypothermia upon acute cold exposure.

To summarize our work, we propose the following working model (Fig. 8). In WT brown adipocytes, DDB1 binds the proximal promoters of early response thermogenic genes including *Ucp1* and *Ppargc1a*. The pre-initiation complex is formed on these promoters, but Pol II is paused under normal conditions. Upon cold exposure, P-TEFb is released from its inhibitory complex and recruited to the proximal promoters of the thermogenic genes by DDB1. P-TEFb then phosphorylates and activates Pol II, resulting in productive transcriptional elongation of the thermogenic genes to maintain body temperature. In DDB1-deficient brown adipocytes, P-TEFb cannot reach the proximal promoters of the thermogenic genes and fails to turn on the transcription of these genes. BAT thus shows a defect in fatty acid oxidation and fails to produce enough heat to main body temperature.

## Discussion

Ever since the discovery of metabolically active BAT in adult humans, BAT has emerged as a potential therapeutic target to treat obesity and related metabolic disorders [3]. Many transcriptional factors and chromatin remodeling factors have been identified to regulate thermogenesis [16, 17], but it remains unclear how the cold signal is integrated with the transcription of thermogenic genes. Here, we show that the early response thermogenic genes are regulated by promoter-proximal pausing of Pol II and that DDB1 plays an essential role in releasing paused Pol II and turning on the transcription of these genes.

The transcription of the thermogenic genes is regulated by various factors [16]. Traditionally, the rate-limiting step of transcription is the formation of the pre-initiation complex. Indeed, histone modifiers such as EHMT1, JMJD1A, and HDAC3 [32, 37, 38] and transcriptional factors such as CREB, ATF2, PRDM16, EBF2, and ZFP516 have also been reported to be required for activation of the thermogenic genes [15, 31, 39, 40]. However, accumulating evidence shows that many immediate early response genes are subjected to regulation by promoter-proximal pausing of Pol II, in which the pre-initiation complex has been formed before stimulation [18]. Here, we show that promoter-proximal pausing of Pol II plays a critical role in cold-induced thermogenesis. Through RNA-Seq and ChIP-Seq analysis, we demonstrate that the transcription of the early response thermogenic genes including *Ucp1* and *Ppargc1a* is controlled by the release of paused Pol II upon cold exposure. Furthermore, blocking the release of Pol II by JQ1 results in cold-induced hypothermia in mice.

DDB1 is typically recognized as a component of the CUL4-E3 complex [27]. We have previously reported a CUL4-independent function in adipogenesis, namely that DDB1 complexes with P-TEFb to initiate the transcriptional cascade of adipogenesis [29]. Here, we identify another CUL4-independent function of DDB1, which is to regulate thermogenesis. The adipose- or BAT-specific *Ddb1* knockout mice exhibited whitened BAT and developed hypothermia when subjected to acute cold exposure. In contrast, knocking out either *Cul4a* or *Cul4b* in adipose tissues has no effect on thermogenesis. Based on the CUL4-independent function of DDB1 in regulating adipogenesis and thermogenesis, we speculate that DDB1 might work independently of CUL4 to regulate other physiological events.

It is notable that disrupting BAT function by depleting DDB1 did not lead to obesity as expected, although BAT activation is associated with lower body mass [11]. Instead, both *Ddb1-AKO* and *Ddb1-BKO* mice showed decreased body weight on HFD, but they were less healthy because both strains showed decreased glucose clearance rate and developed insulin resistance, resembling a phenotype of partial lipodystrophy. These mice also showed a decreased capability for oxidizing fatty acids, elevated plasma levels of free fatty acids, and ectopic lipid storage in the liver. Usually, to maintain body temperature, WAT will upregulate lipolysis to release fatty acids to provide fuels for BAT. However, the *Ddb1-AKO* and *Ddb1-BKO* mice had dramatically decreased expression of the thermogenic genes, and they could not efficiently utilize fatty acids. Consequently, WAT would increase the lipolysis rate, resulting in increased plasma-free fatty acids and decreased size of WAT. The excessive plasma free fatty acids would then be ectopically stored in the liver, leading to fatty liver and insulin resistance. In previous studies, *Ucp1*^*−/−*^ mice are not obese [2], and mice lacking PGC1α in adipose tissues develop insulin resistance without extra weight gain on HFD [41]. Recent human clinical studies show that BAT activation contributes to a small amount of energy metabolism that is unlikely to cause weight loss but improves glucose metabolism [42]. Taken together, BAT activation might be more effective to improve glucose homeostasis than weight loss.

It is also notable that the transcription of *Ucp1* at 22 °C is already lower in the BAT of *Ddb1-AKO* or *Ddb1-BKO* mice than that in the control mice. Ideally, to study the effect of DDB1 on the transcription of *Ucp1*, it would be better to start from the same basal level of *Ucp1*, especially using an inducible knockout system. As we have reported before that inducible knockout of *Ddb1* in *Rosa-Cre*^*ERT2*^–*Ddb1*^*f/f*^ mice causes lethality [29], an adipose-specific inducible knockout model might be more suitable in future studies.

## Materials and Methods

### Materials

We obtained CL316,243, dexamethasone, isobutylmethylxanthine (IBMX), forskolin, isoproterenol, pioglitazone, bovine insulin, urea, sodium dodecyl sulfate (SDS), dithiothreitol (DTT), dimethylsulfoxide (DMSO), and Triton X-100 from Sigma-Aldrich; Dulbecco’s modified Eagle’s medium (DMEM) with low (1 g/L) or high (4.5 g/L) glucose, fetal and neonatal bovine serum, blasticidin, and puromycin from Thermo Fisher Scientific; donkey anti-rabbit IgG or anti-mouse IgG conjugated to horseradish peroxidase from Jackson Immuno Research; protease inhibitor cocktail from Roche Applied Science; and all other chemicals from local suppliers unless otherwise specified.

### Culture, immortalization, and differentiation of BAT adipocytes

For immortalization of BAT preadipocytes, SVFs were isolated from interscapular BAT in newborn *Ddb1*^*f/f*^, *Rosa-Cre*^*ERT2–*^*Ddb1*^*f/f*^, or *AdipoQ-Cre*–*Ddb1*^*f/f*^ mice, cultured and immortalized as previously described [43, 44]. Briefly, cells were cultured in medium A (DMEM high glucose, 20 mM 4-(2-Hydroxyethyl)-1-piperazine ethanesulfonic acid (HEPES) pH 7.4, 10% (v/v) fetal calf serum (FCS), 100 U/ml penicillin, and 100 mg/ml streptomycin) at 37 °C in an atmosphere of 8.8% CO_2_. To differentiate into mature brown adipocytes, cells were cultured to 100% confluence and maintained in medium A for another 2 days. On day 0 of differentiation, cells were treated with medium A containing 1 nM T3, 0.1 μg/ml insulin, 0.125 mM indomethacin, 5 μM dexamethasone, and 0.5 mM IBMX. On days 2, 4, and 6, the medium was changed to medium A containing 1 nM T3 and 0.1 μg/ml insulin. On day 8, fully differentiated brown adipocytes were achieved. To induce deletion of *Ddb1* in SVF-derived brown adipocytes of *Rosa-Cre*^*ERT2*^–*Ddb1*^*f/f*^ mice, cells were treated with 8 μM 4-OHT for 4 days.

### Dual luciferase reporter assay

The CRE reporter was constructed by inserting a 4 × CRE sequence into a pGL3-basic vector (Promega), designated as *CRE-Fluc*. On day 4 of differentiation, cells were treated with 4-OHT (8 μM) to induce deletion of *Ddb1*. On day 6, BAT adipocytes were replated at 4 × 10^4^ cells per well in a 12-well plate. On day 7, cells were transfected with 1.5 μg *CRE-Fluc* and 0.01 μg *Renilla luciferase* (*Rluc*) expressing vector with X-tremeGENE HP (Roche). On day 9, cells were treated with forskolin (10 μM) for 8 h before harvest for dual luciferase reporter assay following the manufacturer’s instructions (Promega, E1960). All the measurements were done in triplicates.

### Retrovirus production and infection

For retrovirus production, HEK293T cells were set up on day 0 at 2.5 × 10^5^ cells per 60-mm dish. On day 2, A-CREB/pMSCV-IRES-GFP II (pMIG II, Addgene, 52107) was co-transfected with pCL-Eco (Addgene, 12371) at 1:1. On day 3, fresh medium was changed. On days 4 and 5, media containing retrovirus particles were collected, centrifuged at 1,500 *g* for 5 min, aliquoted, and stored at −80 °C until use. For retroviral infections, BAT adipocytes were infected with retrovirus in a medium containing 8–10 mg/ml polybrene on days 4 and 6 of differentiation.

### Mice and diets

All mice were housed in colony cages at 22 °C with 12-h light/12-h dark cycles. The dark cycle began at 7 p.m. All animal studies were performed with the approval of the Institutional Animal Care and Research Advisory Committee at Fudan University and Xiamen University.

*Ddb1* ^*f/f*^ mice were generous gifts from Dr. Yong Cang at Shanghai Tech University [26]. *Cul4a*^*f/f*^ mice were generous gifts from Dr. Nengming Xiao at Xiamen University [29]. *Cul4b*^*f/y*^ mice were generous gifts from Dr. Yaoqin Gong at Shandong University [45]. These mice were bred with *AdipoQ-Cre* [46] or *Ucp1-Cre* [47] transgenic mice to generate adipose- or BAT-specific knockout mice.

The chow diet (Xietong Organism, Nanjing, China) contains 12% of calories from fat, 67.4% from carbohydrates, and 20.6% from protein. The HFD (Research Diet, D12492) contained 60% calories from fat, 20% calories from carbohydrate, and 20% calories from protein.

### Histology

Adipose tissues were fixed for 20–48 h in 4% (wt/vol) paraformaldehyde in phosphate buffered saline (PBS). The fixed tissues were embedded in paraffin and sectioned at 5 μm. Slides were then counterstained with H&E. Immunohistochemistry was performed using anti-UCP1 (Abcam, ab10983).

### Electron microscopy

Mice were perfused with 25 ml of a solution containing 4% (wt/vol) paraformaldehyde, 1% (wt/vol) glutaraldehyde, and 250 mM sucrose in 0.1 M cacodylate buffer (pH 7.4), and BAT was isolated and fixed as previously described [48]. The samples were postfixed with 1% (wt/vol) OsO_4_, embedded, and sectioned. Specimens were visualized on a JFC1600 transmission electron microscope.

### Quantification of mitochondrial DNA

BAT was isolated and digested at 55 °C overnight in 0.1 M Tris (pH 8.0), 0.2 M NaCl, 5 mM ethylenediaminetetraacetic acid (EDTA), 0.4% SDS, and 0.2 mg/ml protease K. DNA was phenol-chloroform extracted, precipitated with isopropanol, dried up, and resuspended in 10 mM Tris (pH 8.0) and 1 mM EDTA. DNA was then subjected to qRT-PCR, and the ratio of *Mt-Co1* and *Ndufv1* was used to analyze the relative mitochondrial DNA content [49].

### Gene expression analysis

Total RNA was isolated, and qRT-PCR measurements were performed as described [50]. The primers are listed in Supplementary Table S1. All reactions were done in triplicates. The relative amount of each mRNA was calculated by using the comparative threshold cycle (*C*_*T*_) method. *Cyclophilin* or *36B4* mRNA was used as the invariant control.

RNA-Seq analysis of global gene expression profiling was conducted and analyzed as previously described [29]. Genes with fragments per kilobase per million (FPKM) no less than 1 were included in the analysis.

### Thermo imaging of skin temperature

Mice were genotyped on postnatal day 2. On postnatal day 3 before the onset of hair growth, three pairs of pulps were transferred to a 6-well plate, and skin temperatures were measured by a thermal imaging camera (T300 InfraRed Camera; FLIR Systems).

### Acute cold exposure at 4 °C

Mice were maintained at 22 °C and singly housed a week before the experiment. On the day of experiment, mice were briefly fasted for 4 h and then switched to a 4 °C cold room. Core temperature was measured at 0, 0.5, 1, 2, 3, and 4 h after cold exposure using a digital thermometer with a rectal thermocouple probe (Physitemp, Model BAT-12).

### Metabolic cage study

Metabolic cage analysis was performed in a home-cage system Phenomaster (TSE Systems). Mice were singly housed a week before the experiments and allowed to get acclimated to the metabolic cages for 2 days. Mice were then monitored for 4 days on food intake, body weight, oxygen consumption, carbon dioxide production, and locomotor activities.

To test the effect of CL316,243 on oxygen consumption, each mouse received an intraperitoneal injection of CL316,243 at 10 mg/kg. Oxygen consumption was monitored at 10 min intervals from 30 min before to 3 h after the injection.

### Oxygen consumption analysis of brown adipocytes

OCR was determined at 37 °C using the OROBOROS Oxygraph-2K module (OROBOROS Instruments GmbH). Differentiated brown adipocytes in a 6-cm dish were pretreated with or without 10 μM forskolin for 1 h before the assay. During OCR measurement, cells were subsequentially treated with oligomycin (0.25 μM), phenylhydrazone (FCCP, 5 μM), and Rotenone (0.1 μM). OCR was normalized to the protein content.

### Oral glucose tolerance test and insulin tolerance test

Oral glucose tolerance test and insulin tolerance test were performed as previously described [29]. Briefly, for the oral glucose tolerance test, mice were fasted for 16 h (from 5 p.m. to 9 a.m.) and orally gavaged with 1 or 2 mg/kg glucose as indicated in the figure legends. For insulin tolerance test, mice were briefly fasted for 6 h (from 8 a.m. to 2 p.m.) and intraperitoneally injected with insulin 0.5 or 1 U/kg body weight as indicated in the figure legends. Blood was collected from the tail vein at 0, 15, 30, 60, 90, and 120 min after gavage with glucose or injection with insulin, and blood glucose was measured by a Bayer Contour Glucometer.

### Metabolic parameters

Plasma insulin was measured using a commercial kit (EZassay, MS200). Liver triglyceride and cholesterol were extracted as previously described [51] and measured by commercial kits from Wako Chemicals. To measure triglyceride content in BAT, BAT was homogenized in PBS containing 0.5% SDS, gradually heated up to 95 °C, and kept at 95 °C for another 5 min. The cooled samples were subjected to triglyceride measurement using a commercial kit from Wako Chemicals. The content of triglyceride was normalized to protein content, which was quantified by a Pierce BCA kit (Thermo Fisher Scientific).

### Chromosome immunoprecipitation sequencing

Brown adipocytes and BAT tissue were used for chromosome immunoprecipitation sequencing (ChIP-Seq) analysis. For ChIP with anti-DDB1 antibodies, cells were fixed with 1% glutaraldehyde for 15 min at room temperature. For ChIP with anti-Pol II and anti-Pol II-S2P antibodies, BAT was collected from three mice in each group, grinded in liquid nitrogen, and fixed with 1% formaldehyde for 15 min at 37 °C. Fixation was stopped by adding glycine to a final concentration of 0.14 M and incubating at RT for 10 min, followed by two washes with cold PBS. ChIP, ChIP-Seq library preparation, and data analysis were performed as previously described [29].

### Western blot

Total proteins of adipose tissues were extracted, and Western blot was carried out as previously described. The following antibodies were used: anti-DDB1 (1:50,000, Abcam, ab109027), anti-UCP1 (1:1000, Abcam, ab10983), anti-PPARγ (1:500, Santa Cruz, sc-7273), anti-C/EBPα (1:1000, CST, 8178s), anti-CD36 (1:1000, Sino Biological Inc., 80263-T48), anti-PERILIPIN (1:1000, CST, 9349s), anti-CREB (1:1000, CST, 9197S), anti-pCREB (1:1000, CST, 9198S), anti-COXII (1:1000, Proteintech, 55070-1-AP), anti-COXIV (1:1000, Proteintech, 11424-1-AP), anti-PGC1a (1:1000, Proteintech, 66369-1-Ig), anti-CyclinT1 (1:1000, CST, 81464), anti-CDK9 (1:1000, Santa Cruz, sc-13130), anti-RNA Polymerase II (1:1000, Bethyl, A300-653A), anti-GAPDH (1:5000, Proteintech, 60004-1), and anti-Flag M2 (1:1,000, Sigma, F1804). Membranes were developed in a ChemStudio imaging system (Analytik Jena AG).

### Quantification and statistical analysis

All the statistical analysis was performed using the Student’s two-tailed paired *t*-test. The value represents mean ± SEM. Statistical details of all experiments can be found in the figure legends, including the exact number of cell samples or mice. Asterisks (*) indicate the levels of statistical significance. *P < .05; **P < .01; ***P < .001. No data were excluded from any of the experiments.

## Supplementary Material

loac003_suppl_Supplementary_Material

## Data Availability

The data underlying this article are available in the following datasets: RNA-Seq data: Gene Expression Omnibus GSE192466 (https://www.ncbi.nlm.nih.gov/geo/query/acc.cgi?acc=GSE192466) and Chip-Seq data: Gene Expression Omnibus GSE192457 (https://www.ncbi.nlm.nih.gov/geo/query/acc.cgi?acc=GSE192457).
