# Supplementary material for: DDB1 prepares brown adipocytes for cold-induced thermogenesis
Source: Life Metab. 2022 May 13;1(1):39–53. doi: 10.1093/lifemeta/loac003 (PMC11749000; doi:10.1093/lifemeta/loac003)
Supplement: loac003_suppl_Supplementary_Material [file loac003_suppl_Supplementary_Material.pdf]

DDB1 Prepares Brown Adipocytes for Cold-Induced Thermogenesis

Xu Wang[1,2,‡], Shen-Ying Liu[1,‡], Guo-Sheng Hu[3,‡], Hao-Yan Wang[4], Guo-Liang Zhang[4],

Xiang Cen[4], Si-Ting Xiang[1], Wen Liu[3], Peng Li[1,2,5], Haobin Ye[1], Tong-Jin Zhao[1,2,*]

Figure S1

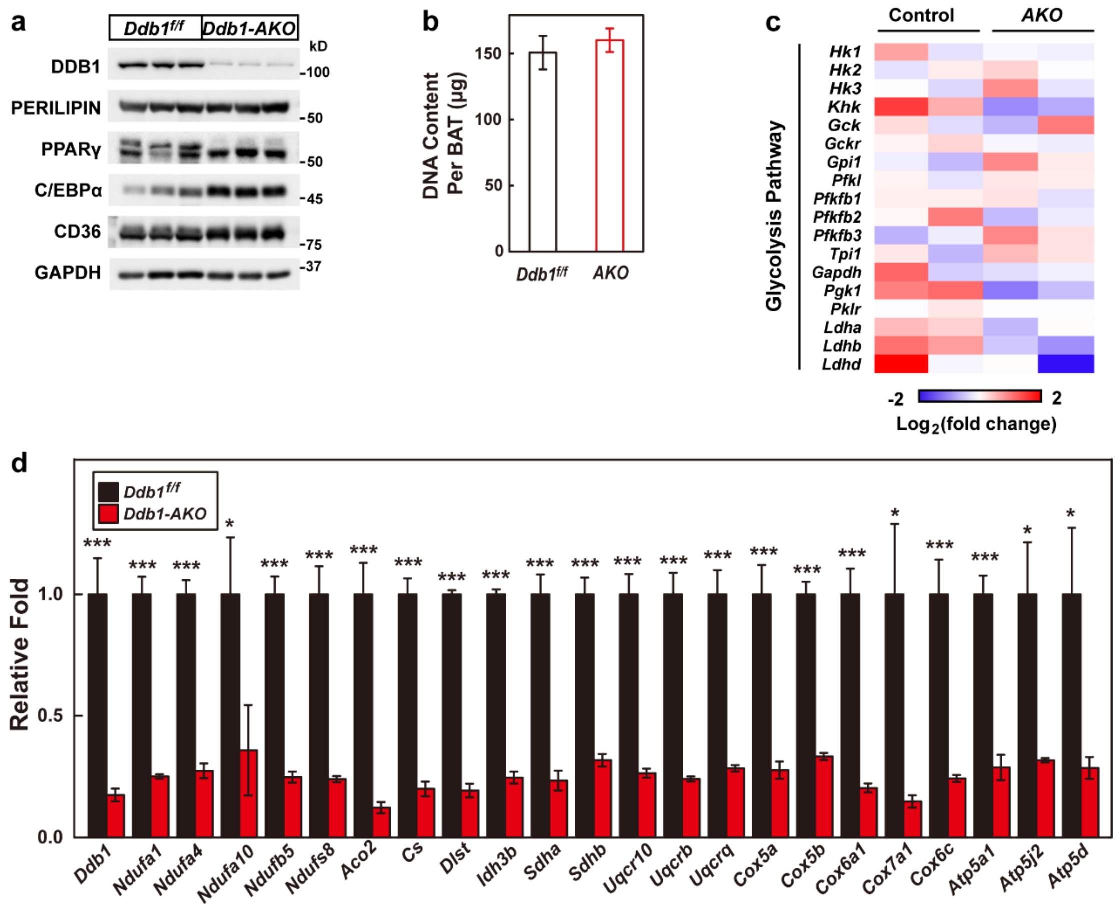

Figure S1. DDB1 is required for the transcription of genes encoding mitochondrial complexes I-V and TCA cycles

(a) Total proteins were extracted from BAT of *Ddb1$^{f/f}$* and *Ddb1-AKO* mice as in Fig. 1a and subjected to western blot using indicated antibodies. **(b)** DNA was extracted from BAT ans quantified. Each value represents mean ± SEM of 3 mice. **(c)** Heat map analysis of glycolysis. The same dataset in Fig. 2 was used for the analysis. **(d)** qRT-PCR analysis of the mRNA levels of indicated genes in BAT of control and *Ddb1-AKO* mice. Samples were prepared as in Fig. 2a. *Cyclophilin* was used as in invariant control. The expression level of each gene in control mice was normalized to 1.0. Each value represents mean ± SEM of 3 mice. Asterisks (*) denote level of statistical significance

(Student's t test) between control and *Ddb1-AKO* mice. *, $p<0.05$; ***, $p<0.001$.

Figure S2

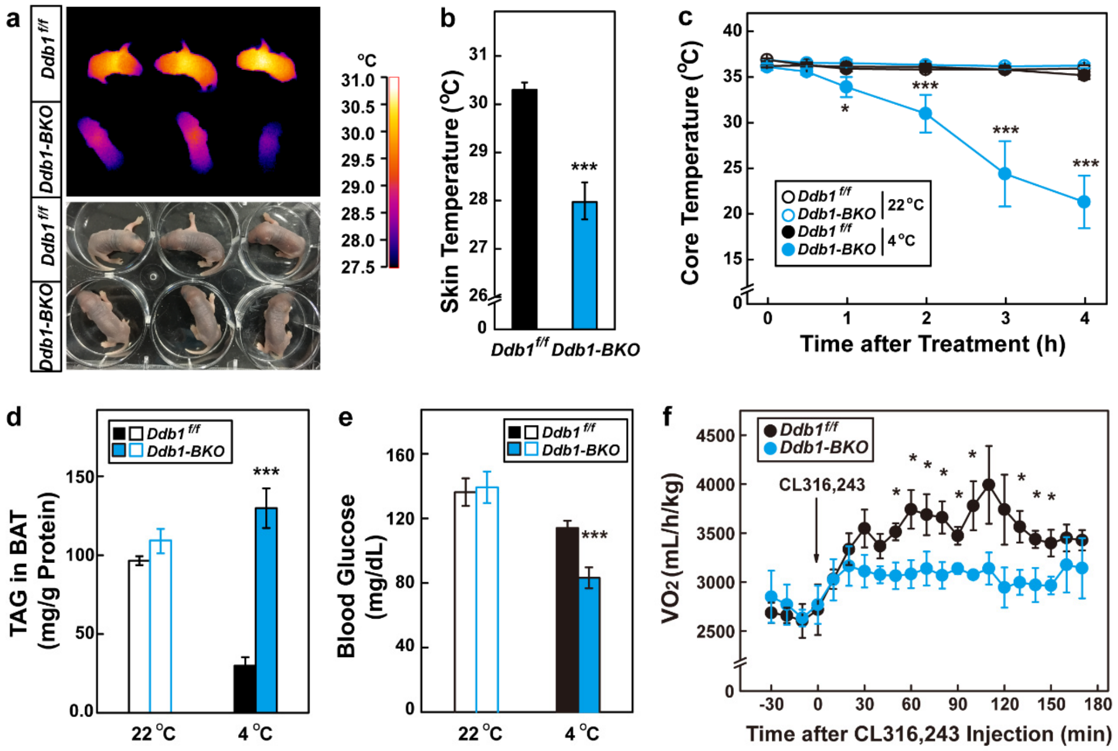

Figure S2. DDB1 is required for cold-induced thermogenesis

Male *Ddb1-AKO* and their control littermates (12-weeks old) were subjected to the same experiments in as Fig. 3a-f. For (a) and (b), 3 pups were used in each group. For (c-f), 6 mice were used for each group. Asterisks (*) denote level of statistical significance (Student's t test). *, p<0.05; ***, p<0.001.

Figure S3

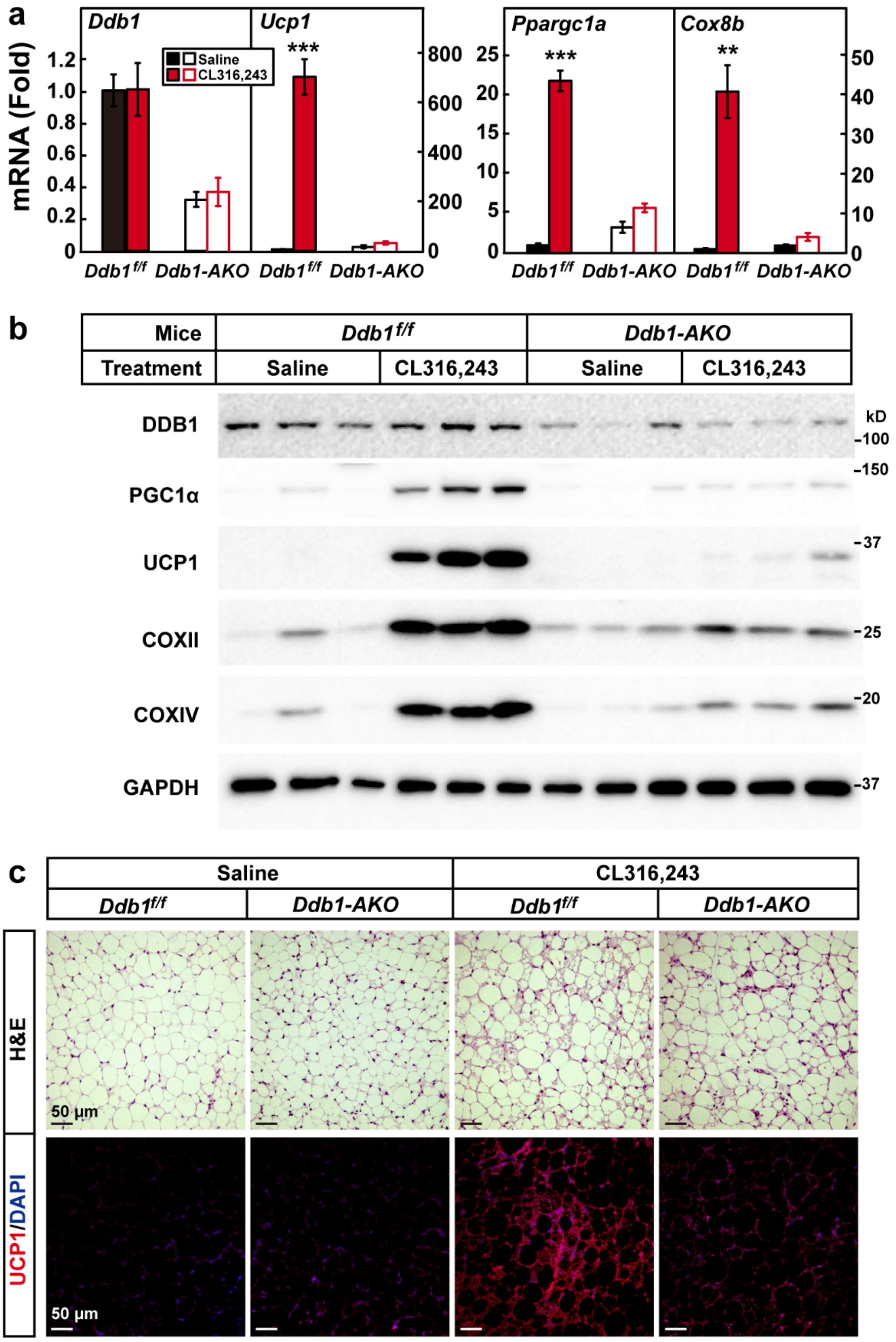

Figure S3. DDB1 is required for browning of iWAT

(a-c) *Ddb1-AKO* and control littermates (8-weeks old, male) received a daily intraperitoneal injection of saline or CL316,243 (1 mg/kg) for 10 days. On day 10, mice

were sacrificed 1 h after the last injection. **(a, b)** Total RNAs and proteins were isolated from inguinal WAT (iWAT) and subjected to qRT-PCR (a) and western blot (b) analysis of the indicated genes. Each value represents mean ± SEM of 3 mice. Asterisks (*) denote level of statistical significance (Student's t test) between CL316,243-injected control and *Ddb1-AKO* mice. **, p<0.01; ***, p<0.001. **(c)** iWAT was subjected to H&E staining and immunostaining of UCP1.

Figure S4

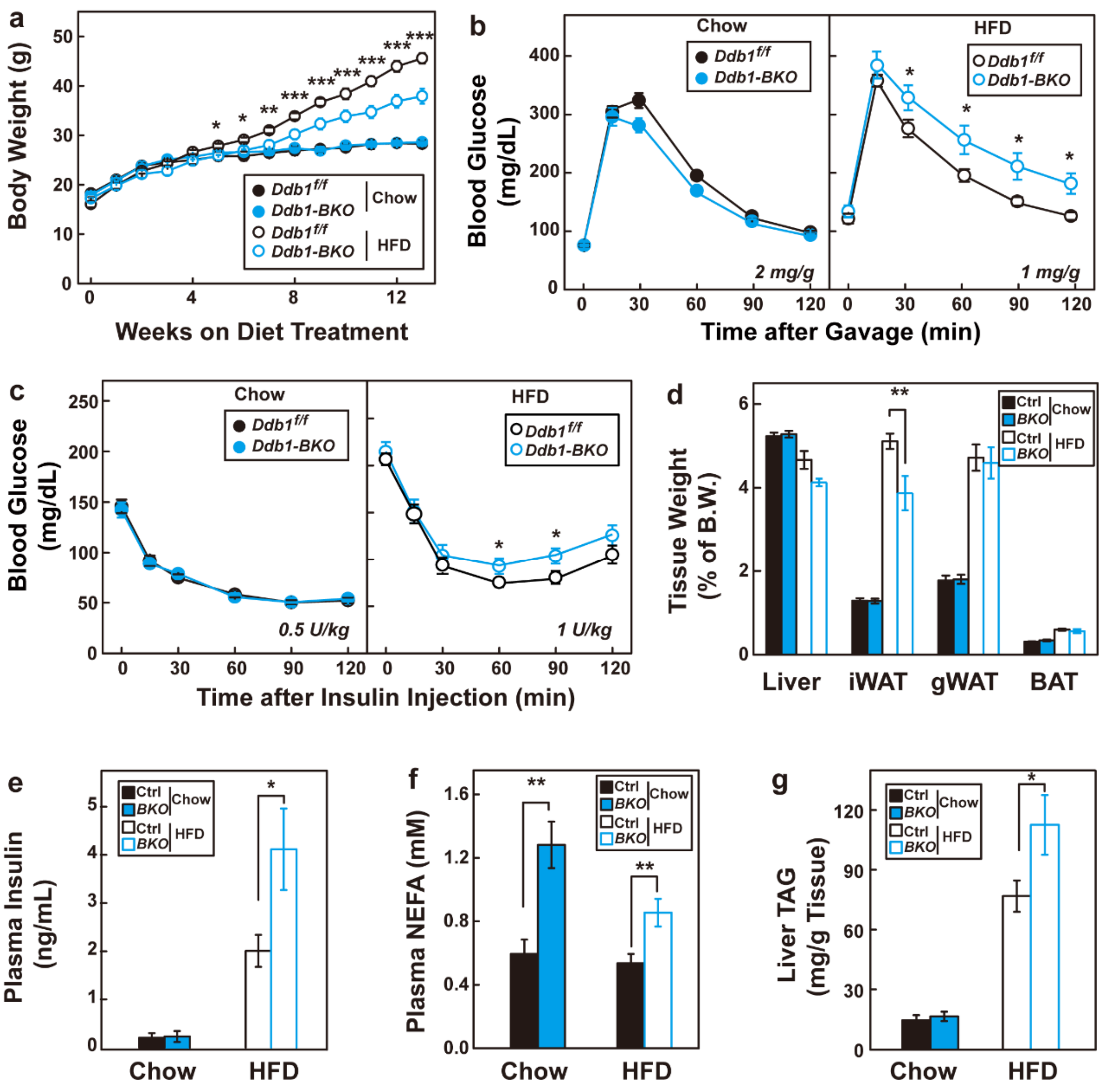

Figure S4. *Ddb1-BKO* mice develop partial lipodystrophy on HFD feeding

(a-g) *Ddb1-BKO* and control littermates (4-weeks old male, n=8 per group) were subjected to chow or HFD feeding for 16 weeks. Body weight (a), glucose tolerance test (b), insulin tolerance test (c), tissue weight (d), plasma insulin (e), plasma free fatty acids (f) and liver triglyceride (g) were measured as in Fig. 4. Each value represents mean ± SEM of 8 mice. Asterisks (*) denote level of statistical significance (Student's t test) between *Ddb1-AKO* and *Ddb1*$^{f/f}$ mice. *, p<0.05; **, p<0.01; ***, p<0.001.

Figure S5

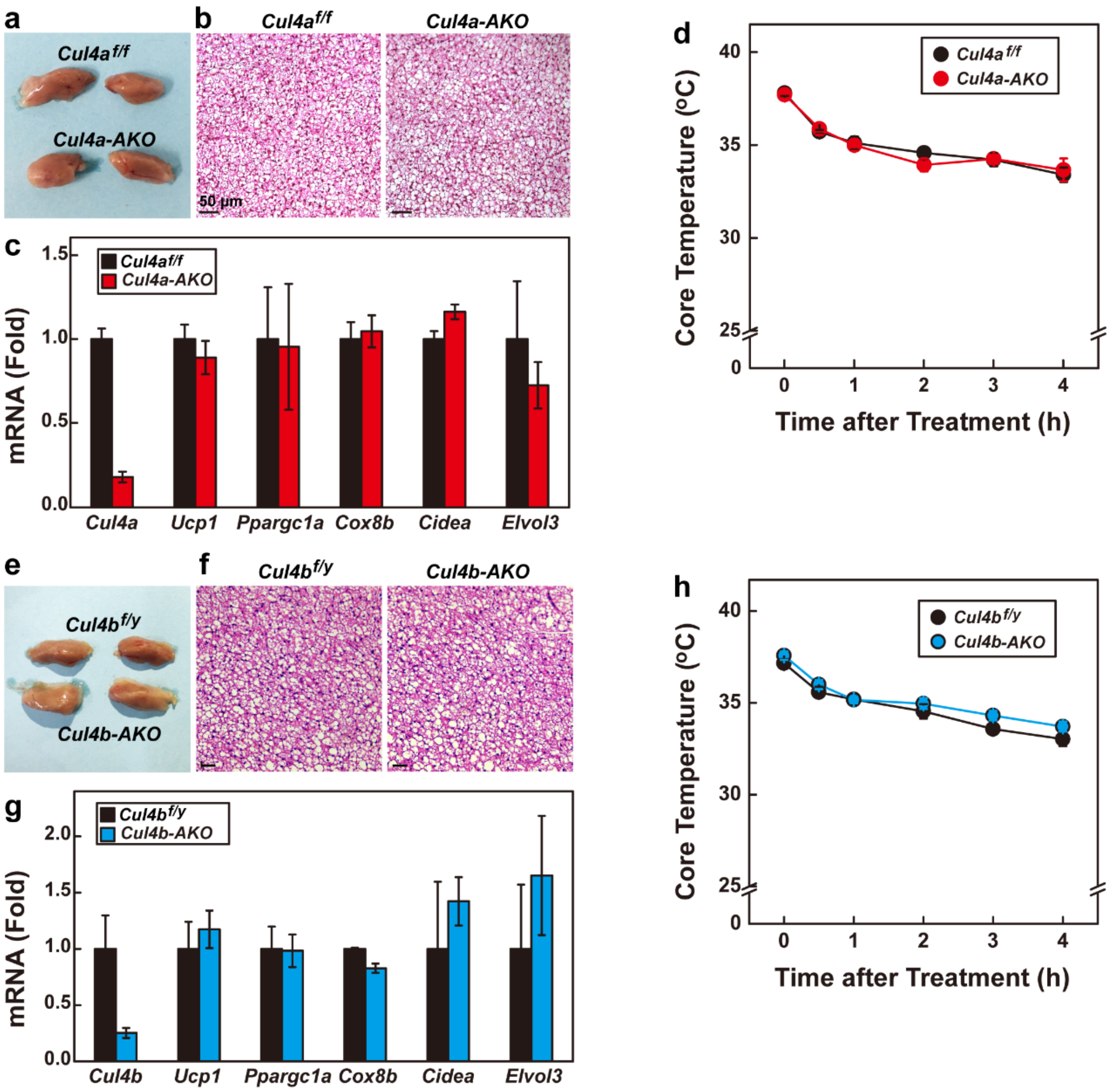

Figure S5. Lack of *Cul4a* or *Cul4b* does not affect thermogenesis

(a, b, e, f) Images and H&E analysis of BAT from *Cul4a*$^{f/f}$ and *AdipoQ-Cre*;*Cul4a*$^{f/f}$ (*Cul4a-AKO*) mice (a, b), or *Cul4b*$^{f/y}$ and *AdipoQ-Cre*;*Cul4b*$^{f/y}$ (*Cul4b-AKO*) mice (e, f). **(c, g)** qRT-PCR analysis of thermogenic genes in *Cul4a*$^{f/f}$ and *Cul4a-AKO* (c), or *Cul4b*$^{f/y}$ and *Cul4b-AKO* mice (g). *Cyclophilin* was used as an invariant control. Each value represents mean ± SEM of 3 mice. **(d, h)** *Cul4a*$^{f/f}$ and *Cul4a-AKO* (d), or *Cul4b*$^{f/y}$ and *Cul4b-AKO* mice (h) were subjected to acute cold exposure at 4°C as in Fig. 2c. Each value represents mean ± SEM of 6 mice.

Figure S6

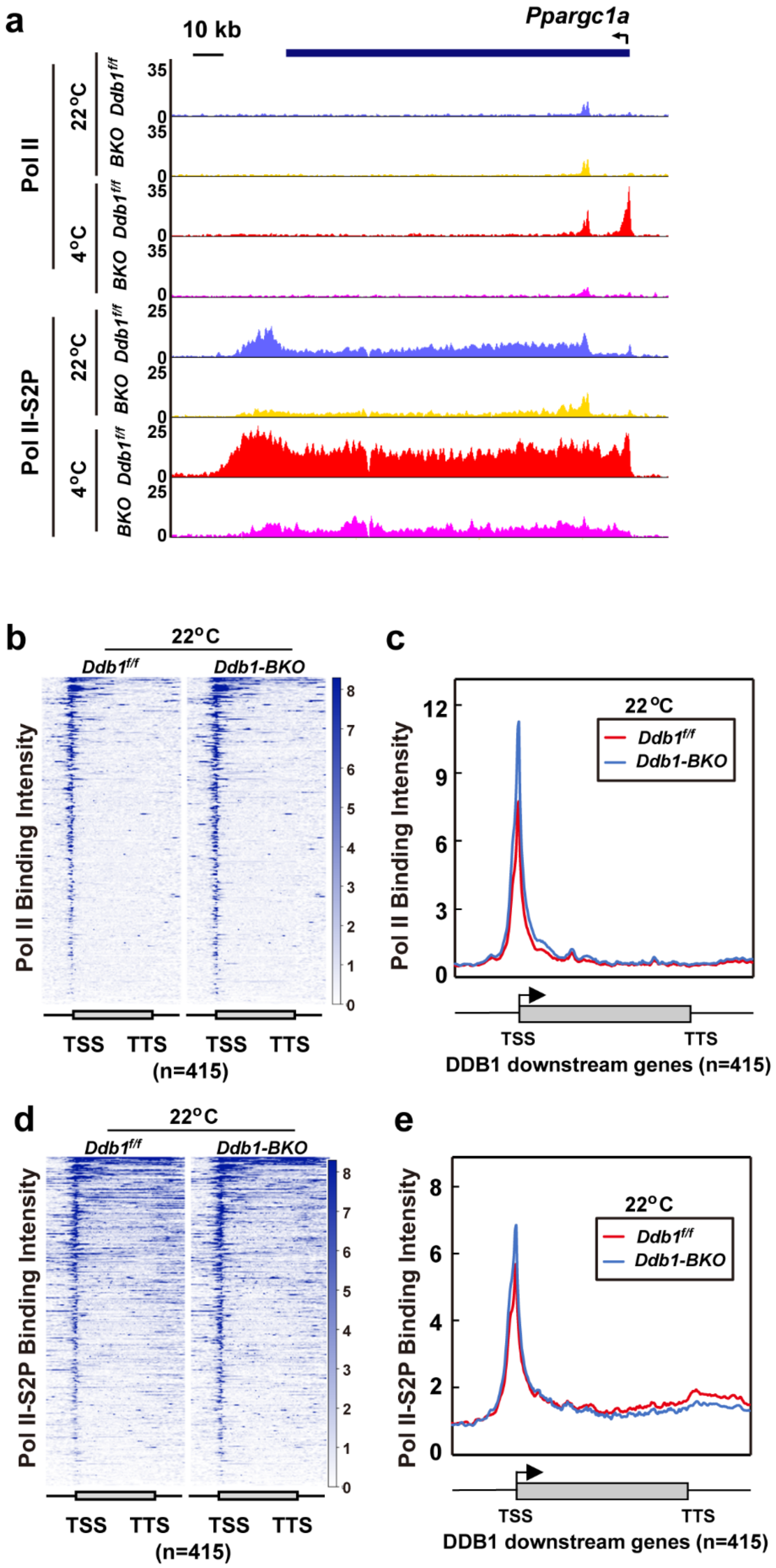

Figure S6. DDB1 promotes the release of paused Pol II on its downstream genes

The same dataset from Fig. 7 were used in the experiment. **(a)** Binding intensity of Pol II and Pol II-S2P on *Ppargc1a* gene in the BAT of *Ddb1*$^{f/f}$ and *Ddb1-BKO* mice at 22°C or 4°C. **(b-e)** Heat map and binding intensity analysis of Pol II (b, c) and Pol II-S2P (d, e) on the 415 downstream genes of DDB1 in the BAT of *Ddb1*$^{f/f}$ and *Ddb1-BKO* mice at 22°C.

Supplemental Table S1: Primers used in the study.

**a. Quantitative real-time PCR Primers**	
mDdb1	CCATTTGCTTTGTCTACCAGG<br>TGGGACTGCTATCACCATTG
mClu4a	TCTCACAAAGTCTCCCCAACG<br>AGGACGTAGGTTCGATCCAGA
mClu4b	TGCTGGCAAAACCACTGTAGG<br>CCAAATGGAGGGTAGCATTGAA
mUcp1	GAGGTGTGGCAGTGTTCATTG<br>GGCTTGCATTCTGACCTTCA
mCidea	TCCTCGGCTGTCTCAATG<br>TGGCTGCTCTTCTGTATCG
mPpargc1a	AACCACACCCACAGGATCAGA<br>TCTTCGCTTTATTGCTCCATGA
mCox8b	TGTGGGGATCTCAGCCATAGT<br>AGTGGGCTAAGACCCATCCTG
mDio2	CAGCTTCCTCCTAGATGCCTA<br>CTGATTCAGGATTGGAGACGTG
mElovl3	TTCTCACGCGGGTTAAAAATG<br>GGGCCTTAAGTCCTGAAACGT
mFgf21	CTGCTGGGGGTCTACCAAG<br>CTGCGCCTACCACTGTTCC
mFfar4	ACCAAGTCAATCGCACCCAC<br>GTGAGACGACAAAGATGAGCC
mEbf2	GGGATTCAAGATACGCTAGGAAG<br>GGAGGTTGCTTTTCAAAATGGG
mCebpb	CGCCTTTAGACCCATGGAAG<br>CCCGTAGGCCAGGCAGT
mPpara	ACAAGGCCTCAGGGTACCA<br>GCCGAAAGAAGCCCTTACAG
mAtf2	CCGTTGCTATTCCTGCATCAA<br>TTGCTTCTGACTGGACTGGTT
mEsrra	GGGGAGCATCGAGTACAGC<br>AGACGCACACCCTCCTTGA
mHdac3	GCCAAGACCGTGGCGTATT<br>GTCCAGCTCCATAGTGGAAGT
mAdrb3	GGCCCTCTCTAGTTCCCAG<br>TAGCCATCAAACCTGTTGAGC
mNdufa1	ATGTGGTTCGAGATTCTCCCT<br>TGGTACTGAACACGAGCAACT
mNdufa4	TCCCAGCTTGATTCCTCTCTT

	GGGTTGTTCTTTCTGTCCCAG
mNdufa10	GAGGTTGCTGAGACTCGTCC<br>CCATCTACTGTTATCACTCGGCT
mNdufb5	CAAGAGACTGTTTGTCGTCAAGC<br>TGTTCACCAGTGTTATGCCAAT
mNdufs8	GGAGAGGAGCGTTGCATTG<br>ATGTCATAGCGTGTCGTTCGG
mAco2	ATCGAGCGGGGAAAGACATAC<br>TGATGGTACAGCCACCTTAGG
mCs	GGACAATTTTCCAACCAATCTGC<br>TCGGTTCATTCCCTCTGCATA
mDlst	CTCGGCACAAGGATGCTTTC<br>GGGCCTTCTCTCCTAGTTCATTA
mIdh3b	TGGAGAGGTCTCGGAACATCT<br>AGCCTTGAACACTTCCTTGAC
mSdha	GGAACACTCCAAAAACAGACCT<br>CCACCACTGGGTATTGAGTAGAA
mSdhb	AATTTGCCATTTACCGATGGGA<br>AGCATCCAACACCATAGGTCC
mUqcr10	CGAGCGAGCCTTCGATCAG<br>ACAGTTTCCCCTCGTTGATGT
mUqcrb	GGCCGATCTGCTGTTTCAG<br>CATCTCGCATTAACCCCAGTT
mUqcrq	CCTACAGCTTGTCGCCCTTT<br>GATCAGGTAGACCACTACAAACG
mCox5a	GCCGCTGTCTGTTCCATTC<br>GCATCAATGTCTGGCTTGTTGAA
mCox5b	TTCAAGGTTACTTCGCGGAGT<br>CGGGACTAGATTAGGGTCTTCC
mCox6a1	TCAACGTGTTCCTCAAGTCGC<br>AGGGTATGGTTACCGTCTCCC
mCox7a1	GCTCTGGTCCGGTCTTTTAGC<br>GTACTGGGAGGTCATTGTCGG
mCox6c	GCGTCTGCGGGTTCATATTG<br>TCTGCATACGCCTTCTTTCTTG
mAtp5a1	TCTCCATGCCTCTAACACTCG<br>CCAGGTCAACAGACGTGTCAG
mAtp5j2	TGCCGAGCTGGATAATGATGC<br>ACCATGCTAATCCCCGAGATG
mAtp5d	TGCTTCAGGCGCGTACATAC<br>CACTTGCTTGACGTTGGCA
**b. mitochondrial DNA Primers**	

mt-Co1	TGCTAGCCGCAGGCATTAC<br>GGGTGCCCAAAGAATCAGAAC
Ndufv1	CTTCCCCACTGGCCTCAAG<br>CCAAAACCCAGTGATCCAGC
